# Acute motor–cognitive responses to a bouldering fatigue protocol in indoor recreational climbers

**DOI:** 10.3389/fphys.2026.1712130

**Published:** 2026-03-05

**Authors:** Bartosz Wilczyński, Mateusz Nowosad, Łukasz Poniatowski, Solene Gerwann, Katarzyna Zorena

**Affiliations:** 1 Department of Immunobiology and Environmental Microbiology, Medical University of Gdańsk, Gdańsk, Poland; 2 Medical University of Gdańsk, Gdańsk, Poland; 3 Department of Physical Culture, Gdańsk University of Physical Education and Sport, Gdańsk, Poland; 4 Institute of Sports and Sports Sciences, Heidelberg University, Heidelberg, Germany

**Keywords:** athlete monitoring, climbing, fatigue, sports physiology, working memory

## Abstract

**Objectives:**

To evaluate the impact of an ecological bouldering fatigue protocol and quantify acute changes in climbing-specific motor performance and visuospatial working memory in indoor climbers.

**Methods:**

Non-randomised pre–post study in 28 indoor boulderers (18 male, 10 females; 15–34 years). Participants attempted sex- and skill-matched problems on a 15° overhanging Kilter Board to volitional exhaustion. Pre- and post-fatigue assessments included finger-hang endurance, pinch-grip strength, explosive pulling power, static balance, upper-limb dynamic stability, and visuospatial working memory.

**Results:**

The protocol achieved all feasibility criteria. The intervention produced physiological stress (heart rate +60 bpm from baseline: 118.9 ± 22.0 to 178.6 ± 11.1; RPE 14.7 ± 1.8) and forearm-related termination in 75%. Performance changes: finger-hang endurance −34.2% (d_z_ = −0.85, p < 0.001); pinch-grip strength −5.8% (d_z_ = −0.53, *p* = 0.009); explosive pulling power −4.8% (d_z_ = −0.52, *p* = 0.010), and visuospatial working memory +16.4% (d_z_ = 0.54, *p* = 0.008). Static balance and upper-limb stability showed trivial non-significant change.

**Conclusion:**

A brief, standardised, sex- and skill-matched bouldering protocol was feasible and induced climbing-specific fatigue. The observed improvement in visuospatial working memory challenges simple fatigue-impairment assumptions. This ecologically valid protocol provides a foundation for future work on motor–cognitive interactions and injury-relevant performance in climbing athletes.

## What is already known on this topic

- Bouldering imposes combined motor and cognitive demands under fatigue, but there is no standardised protocol to assess these domains concurrently.

### What this study adds

- A standardised, skill- and sex-matched bouldering fatigue protocol was feasible, safe, scalable in indoor recreational climbers.
- The protocol reliably induced climbing-specific forearm fatigue while preserving balance and postural control.
- Visuospatial working memory improved post-exercise, suggesting arousal-related facilitation rather than fatigue-related decline.

### How this study might affect research, practice, or policy

- Establishes a reproducible feasibility framework for studying acute motor–cognitive responses in recreational climbers.
- Provides the methodological basis for developing future athlete-level testing and monitoring tools.
- Serves as a necessary first step toward later validation in competitive and elite climbers, where intensified protocols can be evaluated for performance and injury-risk applications.

## Introduction

Bouldering combines explosive strength, technical precision, and rapid decision-making in a time-constrained environment. Despite its inclusion in the Tokyo 2020 Olympics and growing research interest, standardised protocols to assess concurrent motor-cognitive demands under fatigue remain absent (Winkler et al., 2023; Medernach et al., 2025b). Bouldering comprises short (≈10–40 s), technically demanding routes (problems) performed without ropes on low walls; falls are mitigated by mats and spotters, yet acute injuries remain common (Grønhaug et al., 2024; Ehiogu et al., 2025). Indoor bouldering demands strength, flexibility, coordination, and precise movement sequencing, combined with rapid spatial evaluation and tactical decision-making often under time pressure (Saul et al., 2019; Stien et al., 2021; Langer et al., 2023; Medernach et al., 2024).

Fatigue in climbing affects both performance and safety. Physical fatigue mainly targets finger flexors and the shoulder girdle during intense isometric contractions (Phillips et al., 2012) and is a critical predictor of performance, and even advanced climbers showed marked strength loss on 20-mm edges (Padilla-Crespo et al., 2025). While localised forearm fatigue does not impair movement fluidity, suggesting technique remains relatively preserved, it does reduce fall prevention (Valenzuela et al., 2015). Separately, cognitive fatigue—arising from sustained decision-making or prolonged mental load—can also degrade performance, although findings are mixed (Cuchna et al., 2024). Under time pressure, climbers often shift from deliberate analysis to more automatic ‘fast’ decisions, reducing strategic behaviour (Medernach et al., 2025a).

Despite growing research on climbing physiology and performance (Saul et al., 2019; Medernach and Memmert, 2021; Marcolin et al., 2022), little is known about how acute fatigue affects the combined demands of motor execution and cognition. Most studies address these domains separately (Heilmann, 2021), though recent work shows cognitive-behavioural processes (such as previewing and visual search) are crucial, with elite climbers displaying domain-specific proficiency (Medernach et al., 2024; Medernach et al., 2025b; Medernach et al., 2025a). Yet no standardised protocols exist to assess motor–cognitive coupling under fatigue, despite its relevance in training, competition, and outdoor climbing. Such protocols could inform training periodisation, return-to-sport testing, and studies of fatigue-related performance and injury risk. As a first step in the development pipeline, this feasibility study targeted indoor recreational climbers to establish safety, tolerability, and scalability before progressing to validation in competitive athletes.

Visuospatial working memory (WM) is the short-term storage and manipulation of spatial information used to plan movement. In climbing, it supports route previewing, hold selection, body positioning, and updating of planned sequences during execution (Garrido-Palomino et al., 2024). In this study, WM is examined as a post-exertion cognitive outcome rather than as an index of cognitive fatigue, since no dual-task or prolonged mental-load paradigm was used. This distinction is important because physical (peripheral) fatigue and cognitive fatigue arise from different mechanisms (Lorist et al., 2002), and our design specifically tests how climbing-induced exertion modulates visuospatial WM performance outside of a dual-task context, where central resource competition is absent.

Thus, we aimed to determine the impact of a standardised climbing fatigue protocol, and to characterise acute changes in motor (Langer et al., 2023) and cognitive performance in bouldering athletes. We hypothesised the protocol would meet all feasibility criteria and would generate consistent physiological responses across participants.

## Methods

This non-randomised, within-subject, pre–post study was conducted at an indoor climbing facility in Gdańsk, Poland. The primary outcome was protocol feasibility; secondary outcomes were changes in climbing performance (finger-hang, pinch grip, explosive pulling), postural control, lower-limb mobility, and visuospatial working memory. The study complied with the Declaration of Helsinki, received ethics approval from the Bioethics Committee for Scientific Research at the Medical University of Gdańsk (NKB/530/2024), and was prospectively registered (ClinicalTrials.gov: NCT06830655). Written informed consent was obtained; participants <16 years provided assent with parental consent. Reporting followed Template for Intervention Description and Replication (TIDieR guidelines) and CONSORT extension for Pilot and Feasibility Trials (Supplementary Materials 1, 2).

### Participants and recruitment

We recruited indoor recreational boulderers in Gdańsk via a dedicated study website (QR-linked posters at two climbing gyms and one retail store) with online screening and self-scheduling (24 February–15 May 2025). A total of 51 individuals expressed interest in participating in the study. The final analytic sample included 28 participants (18 male, 10 female), ages 15–34 years, classified as intermediate (n = 13) or advanced (n = 15) per International Rock Climbing and Research Association (IRCRA) (Draper et al., 2021). Climbing proficiency was classified using the IRCRA framework following the ‘3:3:3’ rule (Draper et al., 2015): at least three successful ascents of three different problems within the target grade band in the previous 3 months, self-reported via questionnaire (Supp. Mat. S3). Thresholds were Intermediate IRCRA 10–17 (men)/10–14 (women) and Advanced IRCRA 18–23 (men)/15–20 (women).

**FIGURE 1:**
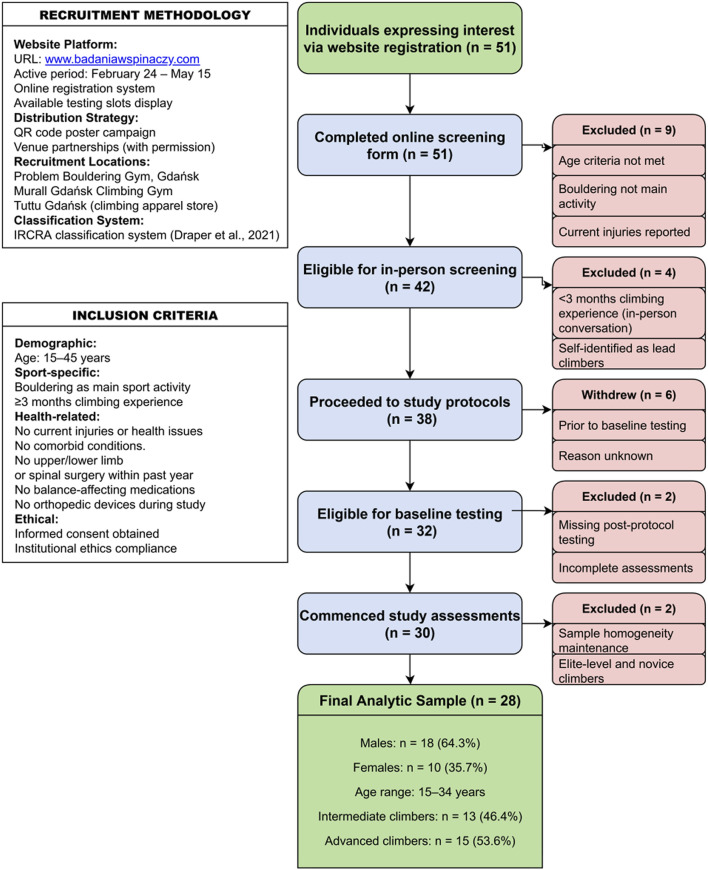
Participant Recruitment Flow Diagram (CONSORT-style flowchart). Note. Flow of participants from initial online registration (n = 51) through screening, eligibility confirmation, and final inclusion (n = 28). Reasons for exclusion are grouped by screening stage. Blue boxes represent retained participants; red boxes indicate exclusions; the green box denotes the final analytic sample stratified by sex and skill level.

Full screening and attrition reasons are shown in the CONSORT-style flow diagram (Figure 1). This phase was intentionally limited to indoor recreational climbers, reflecting the population for whom feasibility and safety must first be established before athlete-grade performance validation.

Inclusion criteria were as follows: age between 15 and 45 years, bouldering as the main sport activity, minimum 3 months of climbing experience, no injuries or health issues during the study period, no comorbid rheumatologic, neurologic, orthopedic, genetic, or cardiovascular conditions, no history of upper/lower limb or spinal surgery within the last year, no medications affecting balance, and no use of orthopedic devices during the study.

### Instruments and measures

After completing the consent form participants underwent face-to-face questionnaires, anthropometric assessments including upper limb length, body mass, and height. A questionnaire was administered to collect demographic data, training volume, and climbing experience (Supplementary Material 3). Climbing advancement level was determined using the IRCRA grading framework (Draper et al., 2021). Participants reported their hardest successfully completed boulder (flash and redpoint), which was mapped to IRCRA grade thresholds to classify them as intermediate or advanced. Participants completed a standardised 6-min dynamic warm-up targeting shoulder–scapular control and finger-flexor activation, designed with a nationally certified route setter. Warm-up was performed before all tests (details in Supplementary Material 4).

Dynamic upper-limb balance was assessed using the standardised UQ-YBT protocol (Gorman et al., 2012). From a push-up position, participants reached with one hand in medial, superolateral, and inferolateral directions along floor-marked lines. After two familiarisation trials per direction, three recorded trials were performed per arm. Reach distances were measured in cm and normalised to arm length (C7 to distal middle fingertip). A composite score was calculated as the mean of the three directions (% arm length). The UQ-YBT demonstrates good–excellent test–retest reliability (ICC 0.80–0.99), low SEM (2.2–2.9 cm), and MDD_95_ 6.1–8.1 cm (Gorman et al., 2012) and 28-day ICCs of 0.91–0.92 have been reported (Westrick et al., 2012). Static balance was assessed with the Stork test (Chaouachi et al., 2014; Obertinca et al., 2018). Participants stood barefoot on the dominant leg, the other foot against the inner knee, hands on hips. On cue, they rose onto the ball of the foot; timing ceased at heel contact, foot movement, or hand removal from hips. Two trials were completed; the longest time was analysed. The test shows responsiveness to training in youth athletes, with effect sizes 0.72–0.92 (Chaouachi et al., 2014).

Lower-limb mobility was assessed with the Climbing-specific Foot Raise in climbing shoes (Draper et al., 2009). From a gym ladder stance, participants raised one leg to maximum height while keeping the contralateral foot and both hands fixed. Height was measured from the starting foothold to the shoe tip at the highest position held for 2 s, guided along a tape without resting or using the beam for support. Vertical displacement (cm) was recorded. The test shows good inter-session reliability (ICC 0.89; r = 0.95–0.99) (Langer et al., 2023).

Pinch strength was measured using a Constant dynamometer (model 14192-709E) per IRCRA guidelines (Assmann et al., 2021). Participants, seated with the elbow at 90°, performed maximal pincer grip between thumb and fingers two–five. Two trials per hand were completed; the highest value (kg) was recorded. Intra-session reliability is excellent (r > 0.99); inter-session correlations vary (r 0.22–0.77), CCC 0.99 (MacKenzie et al., 2020; Langer et al., 2023). The continuous finger hang test was conducted on a 25 mm deep bar (Baláš et al., 2012) using a half-crimp grip without thumb involvement, following the IRCRA protocol. Participants assumed a toe-hanging position with the upper limbs extended overhead, elbows fully extended, hips extended, and knees flexed to 90°, ensuring that the feet were behind the line of the torso and without ground contact. The test was terminated when the participant could no longer maintain the prescribed position. Hang time was recorded in seconds (s), with the best attempt used for analysis. Explosive pulling strength was assessed per IRCRA (Draper et al., 2021). From a dead-hang on a 40 mm edge, participants performed a maximal dynamic pull-up, striking a calibrated board with one hand. Performance (cm) was normalised to arm length. Reliability is excellent: intra-session ICC 0.98 (CV <4.9%), inter-session ICC 0.95–0.98 (CV ≈ 7%) (Langer et al., 2023).

Visuospatial working memory was assessed with the digital 2D Corsi Block-Tapping Test administered via the Psychology Experiment Building Language software (PEBL) (Mueller and Piper, 2014) (https://pebl.sourceforge.net). Testing was performed on a laptop with external mouse in full-screen mode. After three practice trials, participants reproduced illuminated block sequences of increasing length until failing two consecutive sequences at the same length. PRE and POST used non-overlapping, randomly generated sequences. Outcomes included Total Span (sum of correct sequences), Block Span (longest correctly recalled sequence), and Total Correct Trials. Scoring and recording were automated in PEBL, consistent with standard procedures (Whitaker et al., 2020; Heilmann, 2021). Feasibility criteria followed climbing-specific literature and pilot study guidelines (Eldridge et al., 2016; Campbell et al., 2020; Teresi et al., 2022). Prior climbing studies reported consistently high completion (España-Romero et al., 2009; Ferrara et al., 2020). For our novel bouldering protocol, we set conservative thresholds: completion ≥85%, safety <10% minor adverse events, recruitment ≥70%, fidelity ≥90%, and physiological validity ≥80% reaching target forearm fatigue.

### Fatigue protocol

**FIGURE 2:**
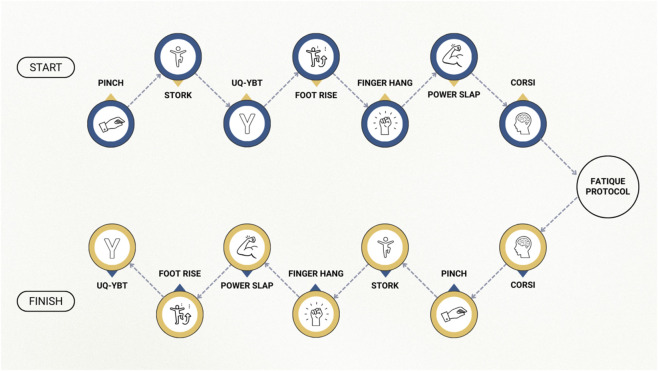
Boulder Route Protocol. From pre-test, through intervention, to post test sequence. Note. Blue icons represent PRE-fatigue assessments; gold icons represent POST-fatigue assessments. The order of tests was reversed post-fatigue to minimise task-order learning effects and to maintain comparable timing relative to the Corsi test. The fatigue protocol (LED-guided bouldering to voluntary exhaustion) was positioned between the two sequences.

**FIGURE 3:**
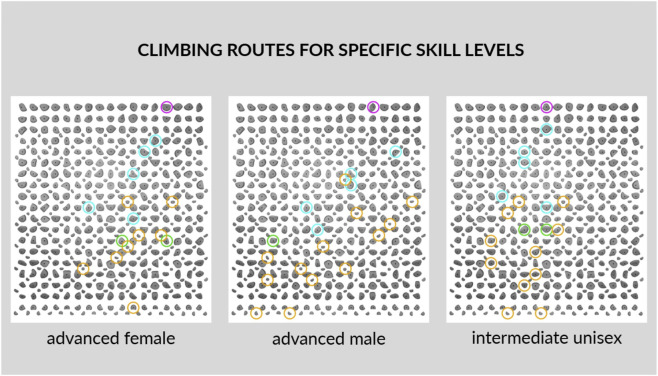
Example of skill-level dependent route configuration used in the fatigue protocol. Note. Three LED-guided routes were used: advanced female (V2), advanced male (V4), and intermediate unisex (V0). Green holds = start; blue = hands-only; yellow = feet-only; purple = top. All designated holds were required during ascent to standardise movement sequence efficiency. The same holds were used for descent to maintain continuous loading.

Climbers performed repeated ascent–descent cycles on a 15° overhanging Kilter Board (Kilter, Boulder, CO, United States) with LED-guided problems and rule-based hold restrictions (hands-only, feet-only, finish hold). Eight pre-set problems were allocated according to skill level and sex. Intermediates of both sexes attempted the same problem (V0, Vermin scale), as IRCRA grading thresholds are identical. Advanced climbers were assigned sex-specific problems (V2 for females, V4 for males, Vermin scale) to reflect IRCRA grade bands. Grades were set by a licensed routesetter and validated through ascent and descent trials, then confirmed by an instructor and two experienced climbers. Participants completed one familiarisation ascent; magnesium carbonate and ≤5 s chalking pauses were allowed. They climbed smoothly and continuously until termination (volitional exhaustion, a sequence of falls indicating inability to complete the assigned moves despite brief re-attempts, or inability to progress). Because falls can occur sporadically, a fixed fall-count threshold was not imposed. Heart rate was monitored via chest strap (Polar, Kempele, Finland); HRstart, HRmax, and HRmean for the final ascent–descent were extracted. Perceived exertion was recorded (Borg RPE 6–20; exploratory CR10). Participants reported limiting muscle groups and termination reason. All attempts were video-recorded for timekeeping and adjudication (route compliance, ascent count). No rest periods were provided other than the brief time required to set up and move between tests (‘transitions’). Inter-test intervals, defined as the elapsed time between completing one test and starting the next, were kept ≤60 s. The test order and protocol are shown in Figure 2. Full operational details (LED coding, device models/firmware, route IDs in Kitler app (example in Figure 3), adjudication criteria) are provided in the Supplementary Material 1 (TIDieR).

### Statistical analyses

Analyses were conducted in Python 3.11 and R 4.3.0, following a pre-specified protocol (TIDieR, STROBE; Supplementary Methods). Assumption testing showed non-normality in Stork (W = 0.729, *p* < 0.001) and Y-Balance (W = 0.889, *p* = 0.006), analysed with both parametric and non-parametric tests. Multivariate normality was violated (Mardia skewness *p* < 0.001), so MANOVA proceeded using Pillai’s trace, robust to such violations. No multivariate outliers were detected. Seven outcomes (pinch, hang, slap, foot rise, Corsi, UQ-YBT, Stork) were analysed with TIME (PRE vs. POST) as a within-subject factor. Pillai’s Trace was the primary statistic; effect sizes were Cohen’s dz with 95% CI, interpreted as negligible (<0.2), small (0.2–0.5), medium (0.5–0.8), or large (≥0.8). Paired t-tests were used for normal data; Wilcoxon signed-rank tests for non-normal. No multiple comparison corrections were applied given exploratory aims. Because the protocol used a sex- and skill-matched design, subgroup analyses were performed descriptively rather than modeled as covariates. Baseline comparability was evaluated using anthropometric, physiological, and climbing-exposure variables. PRE–POST subgroup patterns were examined descriptively to verify whether fatigue responses were consistent across skill level and sex. This feasibility study aimed to recruit ∼30 participants, consistent with pilot guidelines (Billingham et al., 2013; Whitehead et al., 2016). This size was considered adequate to assess feasibility, recruitment, and outcome variability for future trials. Comparable climbing feasibility studies (Valenzuela et al., 2015), n = 14; (Ferrara et al., 2020), n = 15; successfully demonstrated feasibility and physiological changes, supporting our approach. However, to contextualise the planned sample, an indicative GPower calculation for a paired t-test (one-tailed; dz = 0.5; α = 0.05; power = 0.80) suggests a minimum total sample size of n = 27.

### Patient and public involvement statement

Climbing coaches and certified route setters assisted with test selection, warm-up protocols, and problem design by experience level and gender. Grades were verified by instructors and experienced climbers. Gym owners facilitated recruitment by displaying study materials. Community members contributed voluntarily without compensation. The public were not involved in research design, outcome selection, or dissemination planning. Results will be shared via participating gyms and a climbing apparel store.

## Results

All participants reported right-hand dominance and identified bouldering as their primary climbing discipline. According to IRCRA proficiency categories, 15 climbers (53.6%) were classified as Advanced and 13 (46.4%) as Intermediate (Table 1). Mean IRCRA grades were: women–Intermediate 13.83 ± 0.41, Advanced 19.00 ± 2.00; men–Intermediate 15.75 ± 0.46, Advanced 19.40 ± 1.90; overall–Intermediate 15.00 ± 1.04, Advanced 19.29 ± 1.86.

**TABLE 1:** Participant characteristics.

Variables	Mean (SD)	Median (IQR)	Min–Max
Age (years)	24.0 (5.2)	23.0 (20.0–26.0)	15.0–34.0
Body mass (kg)	71.0 (12.1)	68.3 (64.6–80.2)	46.8–98.0
Body height (cm)	175.9 (7.0)	177.5 (170.8–181.0)	163.0–187.0
Body mass index (kg/m^2^)	22.8 (2.6)	22.6 (21.5–24.5)	15.8–28.7
Climbing experience (months)	32.7 (32.2)	17.5 (11.0–38.2)	6.0–102.0
Training frequency (sessions/week)	3.0 (0.8)	3.0 (2.0–4.0)	2.0–4.0

Values are presented as mean (standard deviation) and median (interquartile range).

### Feasibility and protocol response

All primary feasibility criteria were met or exceeded benchmarks (Supplementary Table S1). Protocol completion rate was 93,7% among participants who commenced testing (30/32), with 74.5% recruitment efficiency from initial screening. The protocol demonstrated an excellent safety profile with zero serious adverse events and only minor transient discomfort reported in 10.7% of participants (3/28). Protocol fidelity was high at 96.4% (27/28), with only one participant requiring route modification. Mean perceived exertion (14.7 ± 1.8 RPE) indicated “somewhat hard to hard” intensity. The entire testing session, from questionnaire completion to the final post-test, lasted approximately 90–120 min per participant, including warm-up, familiarisation, and the full test battery.

### Physiological and performance characteristics

The protocol elicited consistent physiological responses and performance patterns across participants (Table 2). Heart rate increased from 118.9 ± 22.0 bpm at start to a maximum of 178.6 ± 11.1 bpm, with sustained high intensity throughout (HRavg: 163.1 ± 14.2 bpm). Participants completed a median of 2 ascents (range: 1–12) over 143.5 s (IQR: 107.3–187.8), though performance varied considerably (72–539 s total). The primary reason for protocol termination was localised forearm fatigue (75% of participants). Secondary reasons included focal discomfort (11%; fingers, shoulder, hand), coordination difficulties (7%), and generalised fatigue (7%).

**TABLE 2:** Physiological responses and performance characteristics during the protocol.

Domain	Variable	Mean (SD)	Median (IQR)	Min–Max
Heart rate	Baseline (bpm)	118.9 (22.0)	120.0 (103.5–130.0)	73–180
Heart rate	Average during protocol (bpm)	163.1 (14.2)	160.5 (157.0–169.8)	129–196
Heart rate	Maximum (bpm)	178.6 (11.1)	177.5 (170.8–184.0)	160–206
Perceived exertion	Borg RPE (6–20)	14.7 (1.8)	15.0 (13.8–16.0)	11–17
Perceived exertion	Borg CR10 (0–10)	5.9 (1.6)	6.0 (5.0–7.0)	3–8
Performance	Completed ascents (n)	3.6 (2.9)	2.0 (2.0–4.3)	1–12
Performance	Total attempts (n)	5.5 (3.1)	6.0 (3.0–7.3)	2–13
Performance	Time to exhaustion (s)	166.8 (95.1)	143.5 (107.3–187.8)	72–539

Values are presented as mean (standard deviation) and median (interquartile range). HR, heart rate; bpm = beats per minute; RPE, Rating of Perceived Exertion (Borg 6–20 scale); CR10 = Borg Category Ratio scale (0–10).

### Global multivariate test of change over time

A repeated-measures MANOVA assessed the effect of time (PRE vs. POST) on seven performance outcomes (pinch grip, finger hang, power slap, foot rise, Corsi total score, UQ-YBT, Stork balance) in 28 participants. The analysis showed a statistically significant multivariate TIME effect, Pillai’s Trace = 0.777, F (7, 21) = 10.44, *p* < 0.0001. The result was consistent across alternative omnibus tests (Wilks’ Λ = 0.223, Hotelling–Lawley Trace = 3.48, Hotelling’s T^2^ = 93.97) indicating a large multivariate effect.

**FIGURE 4:**
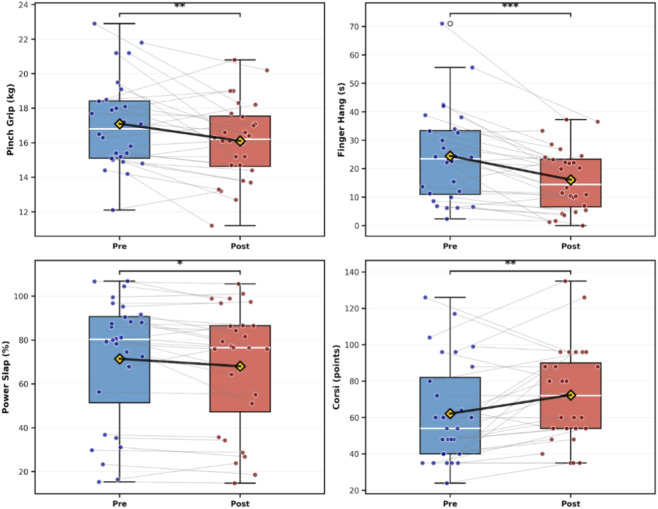
Pre-post changes in significant outcomes with moderate-to-large effect sizes. Note. Boxplots depict PRE (blue) and POST (red) distributions with individual trajectories (grey lines) and group means (yellow diamonds). Significance reflects paired within-subject comparisons (*p < 0.05; **p < 0.01; **p < 0.001).

In the secondary outcome analysis (Table 3; Figure 4), a large reduction was observed in Finger Hang Time (−34.2%, *d*_z_ = −0.85, 95% CI –1.31 to −0.40). Medium effects were found for decreases in Pinch Strength (−5.8%, *d*_z_ = −0.53, 95% CI –0.95 to −0.12) and Power Slap (−4.8%, *d*_z_ = −0.52, 95% CI –0.93 to −0.11), and an increase in Corsi Block Test performance (+16.4%, *d*_z_ = 0.54, 95% CI 0.13–0.96). Small or negligible effects were seen for Stork Balance (*d*_z_ = 0.30), Foot Rise (*d*_z_ = 0.40), and Y-Balance Composite (*d*_z_ = −0.14), with CI crossing zero (Supplementary Figure S1).

**TABLE 3:** Secondary outcome measures for motor skill variables (pre–post).

Variable	Pre, mean (SD)	Post, mean (SD)	Mean change (Post–Pre)	95% CI for mean change	*p*-value	Cohen’s dz	95% CI for dz
Pinch grip strength (kg)	17.10 (2.59)	16.11 (2.30)	−0.99	[−1.717, −0.269]	0.0090**	−0.532	[−0.95, −0.12]
Finger hang test (s)	24.47 (16.34)	16.10 (10.93)	−8.37	[−12.163, −4.568]	0.0001***	−0.854	[−1.31, −0.40]
Power slap test (% arm length)	71.45 (28.76)	68.05 (28.01)	−3.40	[−5.929, −0.871]	0.0103*	−0.521	[−0.93, −0.11]
Corsi block-tapping test (total score)	62.18 (27.98)	72.36 (26.34)	+10.18	[2.904, 17.453]	0.0079**	0.543	[0.13, 0.96]
Climbing-specific foot rise test (cm)	81.62 (7.01)	83.22 (7.02)	+1.593	[0.051, 3.134]	0.0433*	0.401	[−0.00, 0.80]
UQ-YBT composite (% arm length)	99.67 (6.74)	99.14 (7.67)	−0.536	[−2.012, 0.940]	0.4629	−0.141	[−0.53, 0.25]
Stork balance test (s)	7.43 (4.79)	9.70 (10.29)	+2.271	[−0.684, 5.225]	0.1265	0.298	[−0.10, 0.69]

Data are presented as mean ± SD. Δ = mean post–pre change. 95% CI, bias-corrected and accelerated bootstrap confidence interval for the mean change. p-values are from paired t-tests, except for Stork Balance and UQ-YBT, Composite (Wilcoxon signed-rank test; parametric estimates shown for d_z_). d_z_ values represent within-participant standardised mean differences (Cohen’s d for paired designs). Magnitude thresholds: small = 0.2, medium = 0.5, large = 0.8. p < 0.05 was considered statistically significant. Significance stars: p < 0.05 (*), p < 0.01 (**) p < 0.001 (***).

### Exploratory analyses

Post-fatigue Corsi Test performance improved across all metrics (Total Score, Block Span, total correct trials, mean span; all *p* ≤ 0.030; effect sizes *d* = 0.54–0.93). HR measures were strongly intercorrelated (*r* > 0.63, *p* < 0.001) and moderately related to perceived exertion (*r* = 0.40–0.75), with protocol duration strongly associated with number of ascents (*r* = 0.80, *p* < 0.001). No consistent associations were found between physiological load indicators and declines in strength or cognitive outcomes, with small, non-significant correlations predominating. Full statistical outputs and correlation matrices are provided in Supplementary Figure S2 and Supplementary Table S2.

### Subgroup comparisons (skill and sex)

Baseline subgroup comparisons demonstrated comparable anthropometric, physiological, and perceptual load at task onset (all |g| <0.40, *p* > 0.31 for HR and RPE), confirming matching effectiveness (Supplementary Tables S3A, S3B). As expected, advanced climbers exhibited higher climbing-specific capacity (more completed ascents and longer time to exhaustion; r = 0.48), consistent with greater training exposure rather than physiological mismatch. By contrast, sex-based differences in baseline performance were small and inconsistent (|r| ≤0.29). The magnitude and direction of fatigue-induced changes were similar across all subgroups (Supplementary Tables S4A, 4B), with effect sizes for PRE–POST differences falling in comparable ranges for intermediates vs. advanced (dz ≈ −0.5 to −0.9 for strength/capacity declines; dz ≈ 0.2–0.5 for balance/cognitive responses) and for males vs. females.

## Discussion

Our findings indicate the sex- and skill-matched bouldering protocol is feasible, safe, and effective in altering the motor–cognitive profile. The protocol significantly induced localised forearm fatigue without compromising whole-body stability, i.e., large reductions in finger-hang endurance, moderate declines in pinch strength and upper-limb power, a small increase in foot rise mobility, and improved visuospatial working memory, with balance unchanged.

### Feasibility

Feasibility outcomes strongly support the acceptability and practicality of the protocol for indoor bouldering athletes. All participants completed it without serious adverse events, and only 10.7% reported minor transient forearm complaints. Physiological load was high (HRmax 178.6 ± 11.1 bpm; RPE 14.7 ± 1.8; CR10 5.9 ± 1.6), with forearm fatigue causing termination in 75%—consistent with climbing fatigue patterns (Ferrara et al., 2020; Walsh et al., 2023). Of 51 interested athletes, 74.5% were eligible, meeting benchmarks, and the 100% completion rate exceeded prior climbing feasibility studies (España-Romero et al., 2009) and typical pilot thresholds (Eldridge et al., 2016). Together, high recruitment, perfect completion, and low adverse event rates indicate the protocol was well tolerated and ready for larger-scale evaluation.

### Motor performance

Finger-hang endurance showed the largest decline, confirming the protocol’s potency in taxing finger flexor endurance—a critical climbing determinant (Saul et al., 2019; Stien et al., 2019) and a known site of injury (Sjöman et al., 2023). Two participants exhibited extreme reductions, underscoring variability in fatigue tolerance (a complete inability to initiate the post-protocol hang in one female participant (pre = 6.61 s; post = 0 s) and a 94.8% drop in one male participant (pre = 24.23 s; post = 1.25 s)).

Pinch strength fell by 5.8%, consistent with forearm fatigue as a performance limiter (Padilla-Crespo et al., 2025) and prior reports of post-climb swelling and ischemia-related inefficiency (Fryer et al., 2016; Macdonald et al., 2022). Explosive pulling (Power Slap) declined by 4.8%, reinforcing the impact of forearm fatigue on upper-limb power relevant to dynamic manoeuvres (Stien et al., 2021).

In contrast, foot rise improved slightly (+1.6 cm), possibly reflecting compensatory kinematics or potentiation effects. UQYBT and Stork showed no pre–post change, suggesting upper-limb stability and balance are resistant to short grip-dominant fatigue. This contrasts with Han et al. (2025), who found stability gains after warm-up interventions in recreational climbers. Likely explanations include pre-test dynamic warm-up elevating baseline stability scores, the protocol’s primary loading of forearm endurance rather than multi-joint neuromotor control, and ceiling effects given high baseline UQYBT scores (∼99%).

### Cognitive performance

Visuospatial working memory improved across all Corsi subcomponents with medium effects. This is consistent with evidence that acute physical exertion can transiently enhance cognition via arousal and attentional modulation (Ludyga et al., 2016; Wilke and Royé, 2020). Movement-based expertise has also been linked to more efficient visuospatial processing (Barhorst-Cates, 2019), suggesting that task-related sensorimotor engagement may prime spatial memory networks.

A complementary explanation is compensatory recruitment, whereby neural resources are transiently reallocated to stabilise or enhance visuospatial performance following exertion (Tanaka and Watanabe, 2011; Basso and Suzuki, 2016; Herold et al., 2019). This interpretation aligns with neurophysiological models of post-exertional facilitation rather than fatigue-related decline. Although a residual learning effect cannot be fully excluded, the immediate post-test design, absence of dual-task interference, and consistency across subcomponents making compensatory enhancement a plausible and theoretically supported interpretation.

### Subgroup considerations (skill and sex)

The subgroup analyses confirmed that the protocol scaled appropriately across both experience levels and sexes. Although advanced climbers accumulated more ascents and sustained effort longer—a capacity effect consistent with higher training exposure—the relative physiological load at onset and during climbing was comparable across groups, indicating successful matching. Importantly, the direction and magnitude of PRE–POST changes were similar for intermediates and advanced climbers, as well as for males and females, suggesting that the fatigue response was not moderated by subgroup status. Thus, the protocol appears feasible and valid across both training strata and sexes, supporting its applicability for use in mixed climbing cohorts.

### Protocol considerations: standardisation vs. ecological validity

Despite tight apparatus control (fixed 15° Kilter Board, sex/level-matched problems), load varied substantially: time-to-exhaustion ranged 72–539 s (166.8 ± 95.1 s; CV ≈ 57%), and ascents 1–12. This may highlight the technique- and anthropometry-dependent nature of bouldering, where climbers of different body dimensions solve the same sequence with different movement efficiency, even at identical grade difficulty. Individuals with longer limb reach could link moves with fewer repositionings, whereas shorter-limbed climbers required more intermediate contacts. To reduce these efficiency differences, all designated holds were required to be used during the ascent to ensure a consistent number of transitions. A controlled downclimb phase was included to maintain continuous loading and prevent passive rest (i.e., dropping to the mat), thereby standardising time under tension and the conditions leading to termination.

Participants frequently perceived the downclimb as more demanding than the ascent because it offers less momentum and reduced choice of stable gripping positions. While these elements reinforce ecological validity and produce a climbing-specific fatigue pattern, they also introduce protocol-specific loading characteristics that may limit reproducibility. Future refinements may explore fixed-time or density-controlled work bouts, optional downclimb routes, controlled lighting conditions, and intensified wall angles for athlete-level cohorts.

### Implications

The protocol induces climbing-specific forearm fatigue with little impact on balance, offering a platform to study injury-relevant decrements (grip endurance, upper-limb power) alongside cognition. The Corsi improvement raises a testable hypothesis: arousal-mediated enhancement of visuospatial memory after short, intense climbing. Methodologically, future trials should tighten load control and counterbalance cognitive testing to confirm true fatigue effects.

### Limitations

This non-randomised, single-group pre–post design with a small, discipline-specific sample limits generalisability and causal inference. Participant heterogeneity was substantial (bouldering experience 6–102 months, grade V2–V7; BMI 15.8–28.7, including underweight), potentially affecting responses. Despite apparatus control, load standardisation was imperfect (CV ≈ 57%; ascents 1–12), and mandatory downclimbs likely added forearm load. The LED-guided route standardised hold selection but also bypassed route previewing and on-sight decision-making demands, meaning that cognitive responses reflect execution under fatigue rather than naturalistic problem-solving. Potential error sources included demanding test edges (25-mm finger-hang; 40-mm Power Slap) for intermediates. HR measurement via chest-strap relay may still have reduced accuracy. Immediate retest of Corsi without a control or counterbalancing leaves residual learning possible. Finally, only acute responses were assessed; recovery and delayed effects remain unknown. This feasibility study was not powered to model proficiency as a covariate or to test sex-by-skill interactions. Subgroup findings were exploratory. Although experience dispersion was high, IRCRA distributions by sex and group are provided, and pilot-appropriate sensitivity analyses indicate that main within-subject effects remained directionally consistent.

## Conclusion

This standardised bouldering fatigue protocol successfully induced localised forearm fatigue while maintaining high feasibility among indoor recreational climbers (intermediate and advanced boulderers). The unexpected improvement in visuospatial memory following fatiguing exercise challenges the assumption that acute fatigue uniformly impairs cognition and suggests possible arousal-related facilitation. These findings demonstrate that the protocol is scalable across sex and skill subgroups within a recreational climbing population, providing a necessary feasibility foundation in recreational climbers within the development pipeline toward athlete-level validation. Future studies will examine whether the same protocol, or an appropriately intensified version (e.g., steeper angles or higher-grade problems), elicits comparable responses in athlete-level cohorts.

## Data Availability

Data are available in a public, open access repository. The dataset supporting the findings of this study is openly available in the Open Science Framework (OSF) at https://doi.org/10.17605/OSF.IO/HY28U. Route IDs and problem sets can be accessed via the Kilter Board application (links available in Supplementary Material 1).
