# Supplementary material for: Acute motor–cognitive responses to a bouldering fatigue protocol in indoor recreational climbers
Source: Front Physiol. 2026 Mar 5;17:1712130. doi: 10.3389/fphys.2026.1712130 (PMC12999452; doi:10.3389/fphys.2026.1712130)
Supplement: Supplementary file 1 [file Table1.docx]

Supplementary Material

# Supplementary Data

**Supplemental Material 1_TIDieR-Checklist**

**
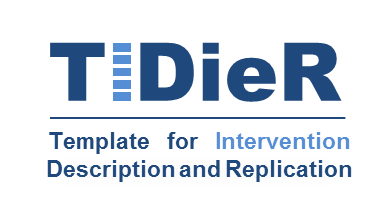
The TIDieR (Template for Intervention Description and Replication) Checklist*:**

Information to include when describing an intervention and the location of the information

| **Item number** | **Item** | **Where located **** | |
| --- | --- | --- | --- |
|  |  | Primary paper  (page or appendix  number) | Other ^†^ (details) |
|  | **BRIEF NAME** |  |  |
| **1.** | *Feasibility and acute motor–cognitive responses to a bouldering fatigue protocol in indoor climbers: a non-randomised pre–post study* | _______0_____ | ______________ |
|  | **WHY** |  |  |
| **2.** | To evaluate the feasibility of an ecological bouldering fatigue protocol and quantify acute changes in climbing-specific motor performance and visuospatial working memory in indoor climbers. | ____1-2____ | _____________ |
|  | **WHAT** |  |  |
| **3.** | Materials: A digitally enhanced climbing training board – Kilter Board – have been used to perform fatigue protocol on it. Boulders used in the study are available on kilter board app under the titles “Intermediate research route” – used for intermediate climbers, “advanced female research route” – used for female advanced climbers and “advanced male research route” used for male advanced climbers. The Kilter Board was set at an overhang angle of +15 degrees for intermediate as well as advanced climbers. | ______3-5___ | Kilter board app: <https://play.google.com/store/apps/details?id=com.auroraclimbing.kilterboard&pcampaignid=web_share>  Kilter board app for apple: <https://apps.apple.com/pl/app/kilter-board/id1215919336?l=pl> |
| **4.** | Participants were instructed on each stage of the fatigue protocol. First, they were introduced to the Borg Rating of Perceived Exertion (RPE) scales, which they would later use to assess their level of fatigue after completing the protocol.  Next, each participant performed one familiarization trial consisting of a single ascent and descent on a bouldering route set on a Kilter Board. The route was selected according to the participant’s climbing level. After the trial climb, participants proceeded directly to the fatigue protocol.  During the climbs, holds were illuminated in different colors to indicate their purpose: green holds marked the starting grips (both hands placed on green holds at the beginning), blue holds were designated for hands only, yellow holds for feet only, and the purple hold indicated the top (final hold).  Participants were instructed to climb as quickly and smoothly as possible, using all available holds designated for their route. After touching the top hold with both hands, they immediately descended along the same holds until reaching the starting position again (both hands on the green start holds). The next ascent then began immediately.  This cycle of climbing up and down was repeated continuously until the participant experienced exhaustion that prevented further climbing. Short breaks (e.g., for chalking up or briefly shaking out the arms) were allowed but could not exceed 5 seconds at a time.  In the event of an unintended fall (due to a slip, misstep, or poor grip unrelated to fatigue), participants were instructed to immediately restart the climb from the starting holds.  Upon completion (i.e., when the participant could no longer continue), they were asked to rate their fatigue on the Borg scale and to provide a brief explanation of the reason for stopping.  The protocol was recorded on video to monitor fidelity of execution and to calculate the duration of the protocol, which was measured from the moment the last limb left the ground to the final loss of contact with the climbing wall. | _______4-5_____ | _____________ |
|  | **WHO PROVIDED** |  |  |
| **5.** | Routesetter – National Licenced Routesetter, one of the Problem Bouldering owners, outdoor and indoor boulder higher elite climber.  During the execution of the protocol, one of the co-authors—a fifth-year physiotherapy student at the Medical University of Gdańsk (GUMed), a certified paramedic and intermediate boulder climber— and another co-author, a physiotherapist and PhD candidate at the Academy of Physical Education and Sport in Gdańsk (AWFiS) were responsible for explaining the procedure to participants, monitoring rest intervals, recording the climbing sessions, and ensuring safety. | _______3-5_____ | _____________ |
|  | **HOW** |  |  |
| **6.** | Face-to-face provided individually | ________3____ | _____________ |
|  | **WHERE** |  |  |
| **7.** | Climbing wall – Problem Bouldering Gdańsk | _______3____ | _____________ |
|  | **WHEN and HOW MUCH** |  |  |
| **8.** | The fatigue protocol was carried out once for each participant, after completing all pre-protocol functional tests and immediately following the Corsi Block-Tapping test. They climbed smoothly and continuously until termination (exhaustion, repeated falls, or inability to progress). | _______4_____ | _____________ |
|  | **TAILORING** |  |  |
| **9.** | The bouldering problems used in the study were custom-designed and tailored to the climbers' individual skill levels. Each route was created to represent the lowest difficulty level within the corresponding experience category, based on the climbing skill classification scale and difficulty grades recommended by the International Rock Climbing Research Association. For intermediate climbers, a V0-level problem (according to the Vermin scale) was created. For advanced female climbers, a V2 route was designed, and for advanced male climbers, a V4 route was used. | _______3-4_____ | IRCRA Grading Scale: <https://ircra.rocks/reporting-grades-in-climbing-research/> |
|  | **MODIFICATIONS** |  |  |
| **10.^ǂ^** |  | N/A | _____________ |
|  | **HOW WELL** |  |  |
| **11.** | Planned: Fidelity of execution was monitored by two co-authors of the study. One of them additionally recorded each fatigue protocol session. | ___7-8_______ | Supplemental Table 1: Feasibility Outcomes |
| **12.^ǂ^** | Actual: All participants completed the fatigue protocol. However, most participants experienced difficulties during the descent phase of the selected routes. Additionally, the majority of protocols were terminated not due to general fatigue, but rather due to localized forearm fatigue. | _____7-8_______ |  |

**Supplemental Material 2_CONSORT-extension-Pilot-and-Feasibility-Trials-Checklist**


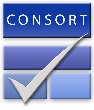
CONSORT 2010 checklist of information to include when reporting a pilot or feasibility trial*

| Section/Topic | Item No | Checklist item | Reported on page No |
| --- | --- | --- | --- |
| Title and abstract | | | |
|  | 1a | Identification as a pilot or feasibility randomised trial in the title | *“Acute motor–cognitive responses to a bouldering fatigue protocol in indoor recreational climbers”* |
|  | 1b | Structured summary of pilot trial design, methods, results, and conclusions (for specific guidance see CONSORT abstract extension for pilot trials) | Objectives/ Methods / Results/ Conclusion |
| Introduction | | | |
| Background and objectives | 2a | Scientific background and explanation of rationale for future definitive trial, and reasons for randomised pilot trial | the first three paragraphs of the Introduction section |
|  | 2b | Specific objectives or research questions for pilot trial | the last paragraph of the Introduction section |
| Methods | | | |
| Trial design | 3a | Description of pilot trial design (such as parallel, factorial) including allocation ratio | Methods – first paragraph |
|  | 3b | Important changes to methods after pilot trial commencement (such as eligibility criteria), with reasons | Methods – Participants and recruitment (first two paragraphs) |
| Participants | 4a | Eligibility criteria for participants | Methods – Participants and recruitment (last paragraph) |
|  | 4b | Settings and locations where the data were collected | Methods – Participants and recruitment (first two paragraphs) |
|  | 4c | How participants were identified and consented | Methods – Participants and recruitment (first two paragraphs) |
| Interventions | 5 | The interventions for each group with sufficient details to allow replication, including how and when they were actually administered | Methods – Fatigue Protocol |
| Outcomes | 6a | Completely defined prespecified assessments or measurements to address each pilot trial objective specified in 2b, including how and when they were assessed | Methods – Instruments and Measures |
|  | 6b | Any changes to pilot trial assessments or measurements after the pilot trial commenced, with reasons | - |
|  | 6c | If applicable, prespecified criteria used to judge whether, or how, to proceed with future definitive trial | - |
| Sample size | 7a | Rationale for numbers in the pilot trial | Methods – Statistical Analyses |
|  | 7b | When applicable, explanation of any interim analyses and stopping guidelines | - |
| Randomisation: |  |  |  |
| Sequence  generation | 8a | Method used to generate the random allocation sequence | - |
|  | 8b | Type of randomisation(s); details of any restriction (such as blocking and block size) | - |
| Allocation  concealment  mechanism | 9 | Mechanism used to implement the random allocation sequence (such as sequentially numbered containers), describing any steps taken to conceal the sequence until interventions were assigned | - |
| Implementation | 10 | Who generated the random allocation sequence, who enrolled participants, and who assigned participants to interventions | - |
| Blinding | 11a | If done, who was blinded after assignment to interventions (for example, participants, care providers, those assessing outcomes) and how | - |
|  | 11b | If relevant, description of the similarity of interventions | - |
| Statistical methods | 12 | Methods used to address each pilot trial objective whether qualitative or quantitative | Methods – Instruments and Measures |
| Results | | | |
| Participant flow (a diagram is strongly recommended) | 13a | For each group, the numbers of participants who were approached and/or assessed for eligibility, randomly assigned, received intended treatment, and were assessed for each objective | Figure 1. Participant Recruitment Flow Diagram (CONSORT-style flowchart) |
|  | 13b | For each group, losses and exclusions after randomisation, together with reasons | - |
| Recruitment | 14a | Dates defining the periods of recruitment and follow-up | Methods – Participants and recruitment (first two paragraphs) |
|  | 14b | Why the pilot trial ended or was stopped | - |
| Baseline data | 15 | A table showing baseline demographic and clinical characteristics for each group | Results Table 1 |
| Numbers analysed | 16 | For each objective, number of participants (denominator) included in each analysis. If relevant, these numbers  should be by randomised group | Results |
| Outcomes and estimation | 17 | For each objective, results including expressions of uncertainty (such as 95% confidence interval) for any  estimates. If relevant, these results should be by randomised group | Results |
| Ancillary analyses | 18 | Results of any other analyses performed that could be used to inform the future definitive trial | Results |
| Harms | 19 | All important harms or unintended effects in each group (for specific guidance see CONSORT for harms) | Results |
|  | 19a | If relevant, other important unintended consequences | - |
| Discussion | | | |
| Limitations | 20 | Pilot trial limitations, addressing sources of potential bias and remaining uncertainty about feasibility | Discussion – Limitation section |
| Generalisability | 21 | Generalisability (applicability) of pilot trial methods and findings to future definitive trial and other studies | Discussion - Implications |
| Interpretation | 22 | Interpretation consistent with pilot trial objectives and findings, balancing potential benefits and harms, and  considering other relevant evidence | Discussion |
|  | 22a | Implications for progression from pilot to future definitive trial, including any proposed amendments | Discussion - Implications |
| Other information | | |  |
| Registration | 23 | Registration number for pilot trial and name of trial registry | prospectively registered (ClinicalTrials.gov: NCT06830655) |
| Protocol | 24 | Where the pilot trial protocol can be accessed, if available | openly available in the Open Science Framework (OSF) at https://doi.org/10.17605/OSF.IO/HY28U |
| Funding | 25 | Sources of funding and other support (such as supply of drugs), role of funders | The publication was co-financed by the state budget under the program of the Polish Minister of Education and Science under the name “Excellent Science”, project no. DNK/SP/548321/2022. |
|  | 26 | Ethical approval or approval by research review committee, confirmed with reference number | The study received prior approval from the Independent Bioethics Committee – Medical University of Gdańsk (application number: NKBBN/241/2023) |

Citation: Eldridge SM, Chan CL, Campbell MJ, Bond CM, Hopewell S, Thabane L, et al. CONSORT 2010 statement: extension to randomised pilot and feasibility trials. BMJ. 2016;355. This is an Open Access article distributed in accordance with the terms of the Creative Commons Attribution (CC BY 3.0) license (<http://creativecommons.org/licenses/by/3.0/>), which permits others to distribute, remix, adapt and build upon this work, for commercial use, provided the original work is properly cited.

**Supplemental Material 3_Questionnaire**

**PERSONAL QUESTIONNAIRE**

**PARTICIPANT DATA**

*Name:* ___________________________

*Participant Number:* ___________________________

*Date and Time:* ___________________________

*Date of Birth:* ___________________________

*Age:* ___________________________

*Gender:* ___________________________ *Male / Female / Other*

**HEIGHT AND WEIGHT**

*Height (cm):* ___________________________

*Body Weight (kg):* ___________________________

*Dominant Hand:* ___________________________ *Right / Left*

**CLIMBING EXPERIENCE**

What is your main sport discipline?

How much time do you dedicate to warm-up before climbing training?

How long have you been practicing bouldering? (in years and months)

How many times per week do you practice bouldering?

How long does one training session take you?

(F = Flash; RP = Red-point)

| **Period** | **F** | **RP** |
| --- | --- | --- |
| Hardest route completed in the last 3 months | _____ | _____ |
| Hardest route completed in the last 12 months | _____ | _____ |
| Hardest route completed in entire climbing career | _____ | _____ |

Have you ever sustained a climbing-related injury that prevented training or work for at least 12 hours?

**YES** **NO**

If yes:

How many such injuries have you sustained?

Which body parts were affected by injuries and what was the severity of the injury? (Please circle selected answers)

*(Injury Severity:

- mild: no medical intervention required
- moderate: outpatient therapy, no permanent damage
- severe: hospitalization, surgical intervention, healing with permanent damage
- critical: life-threatening danger, multi-organ trauma)*

| **Body Part** | **Injury Severity** |
| --- | --- |
| HEAD / FACE | mild / moderate / severe / critical |
| NECK / CERVICAL SPINE | mild / moderate / severe / critical |
| SHOULDERS / CLAVICLE | mild / moderate / severe / critical |
| UPPER ARM | mild / moderate / severe / critical |
| ELBOW | mild / moderate / severe / critical |
| FOREARM | mild / moderate / severe / critical |
| WRIST | mild / moderate / severe / critical |
| HAND/FINGERS/THUMB | mild / moderate / severe / critical |
| CHEST | mild / moderate / severe / critical |
| THORACIC SPINE | mild / moderate / severe / critical |
| ABDOMEN | mild / moderate / severe / critical |
| LUMBAR SPINE | mild / moderate / severe / critical |
| PELVIS / BUTTOCKS | mild / moderate / severe / critical |
| HIP / GROIN | mild / moderate / severe / critical |
| THIGH | mild / moderate / severe / critical |
| KNEE | mild / moderate / severe / critical |
| LOWER LEG | mild / moderate / severe / critical |
| ANKLE | mild / moderate / severe / critical |
| FOOT / TOES | mild / moderate / severe / critical |

**Supplemental Material 4_Warm-up**

**Supplementary Material 4: Standardized dynamic warm-up protocol.**

| **Phase** | **Component** | **Exercise/Activity** | **Duration** | **Instructions** |
| --- | --- | --- | --- | --- |
| 1 | Full-body mobility | Head circles | 20 s | Complete range of motion |
|  |  | Shoulder circles | 20 s | Forward and backward direction |
|  |  | Pelvis circles | 20 s | Maintain neutral spine |
| 2 | Scapular activation | Scapular push-ups | 30 s | Push-up position, protract/retract scapulae |
|  |  | Shoulder taps | 30 s | Maintain plank position, alternate arms |
| 3 | Prone shoulder engagement | Prone swimmers | 30 s | Arms extended, alternate lifting |
|  |  | Half reversed snow angels | 30 s | Prone position, controlled movement |
| 4 | Standing band work | Y-raises | 20 s | Arms form "Y" shape overhead |
|  |  | T-raises | 20 s | Arms parallel to ground |
|  |  | A-raises | 20 s | Arms form "A" shape |
|  |  | External rotation | 15 s per limb | 90° shoulder abduction |
|  |  | Internal rotation | 15 s per limb | 90° shoulder abduction |
| 5 | Finger-flexor priming | Progressive hangs | 3 × 7 s | 25-40 mm edge, 3 s rest between |
|  |  |  | Hang 1 | Full lower-limb support |
|  |  |  | Hang 2 | Light toe contact only |
|  |  |  | Hang 3 | No lower-limb support |
| 6 | Transition | Rest period | ≤30 s | Brief transition before testing |

***Total Duration:*** *Approximately 6 minutes.* ***Equipment:*** *Resistance band (medium resistance), Fixed climbing edge (25-40 mm depth). Supervisor ensures full elbow extension during hangs, Maintain neutral spine positioning throughout, Terminate protocol if pain or adverse symptoms occur. Developed in collaboration with nationally certified route setter, targeting shoulder-scapular control and finger-flexor activation specific to climbing performance testing.*

# Supplementary Figures and Tables

For more information on Supplementary Material and for details on the different file types accepted, please see [here](https://www.frontiersin.org/guidelines/author-guidelines#supplementary-material).

## Supplementary Figures

**Supplemental Figure 1**


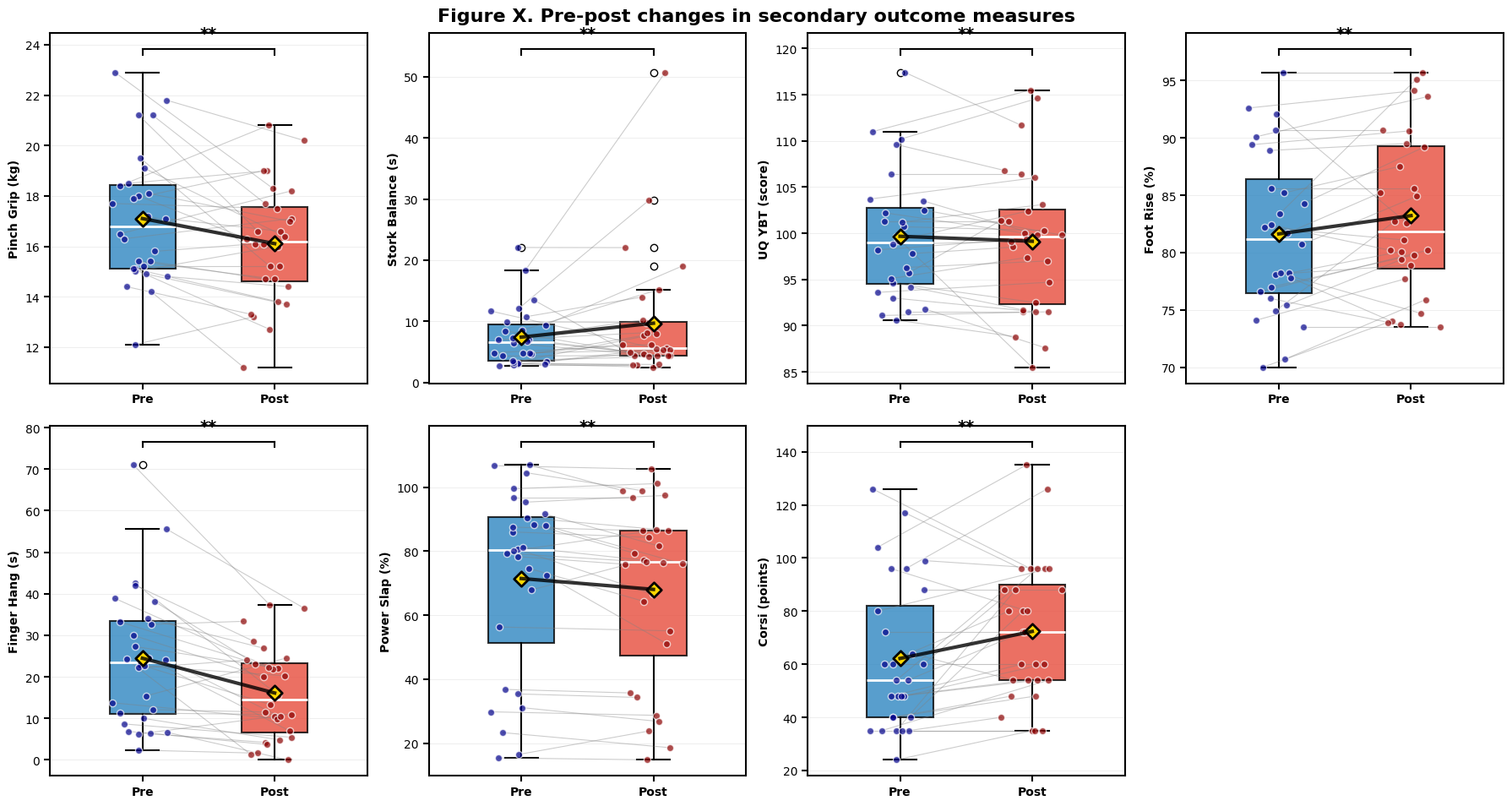


***Legend:*** *Box-and-whisker plots showing individual participant responses (connected dots), group medians (box lines), and means (gold diamonds) for climbing performance and cognitive measures before and after the fatigue protocol (N = 28). Significance brackets indicate statistical comparisons: * p < 0.05, ** p < 0.01, *** p < 0.001. Effect sizes (Cohen's dz): Finger Hang = -0.854, Corsi = 0.543, Pinch Grip = -0.532, Power Slap = -0.521.*

**Supplemental Figure 2**. Correlation analysis of fatigue measurements.


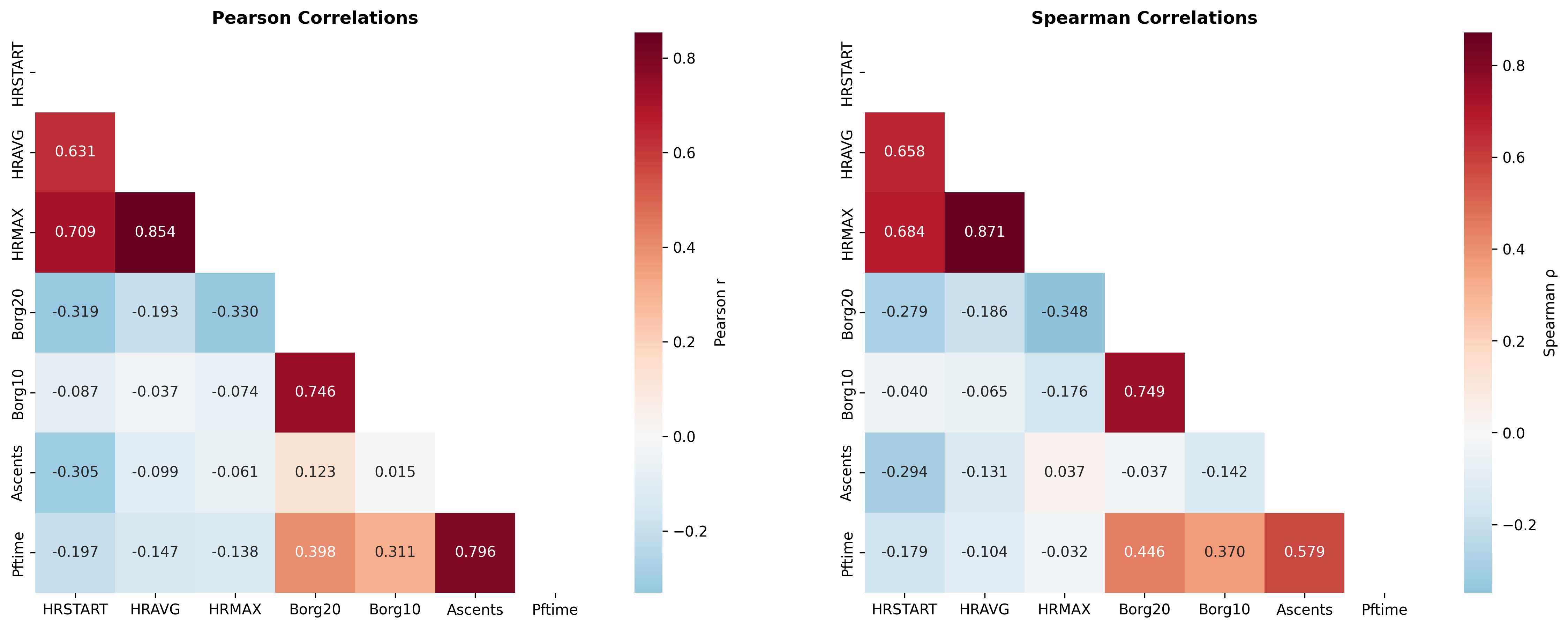


**Supplemental Figure 3**. Examples of participants during the fatigue climbing protocol.


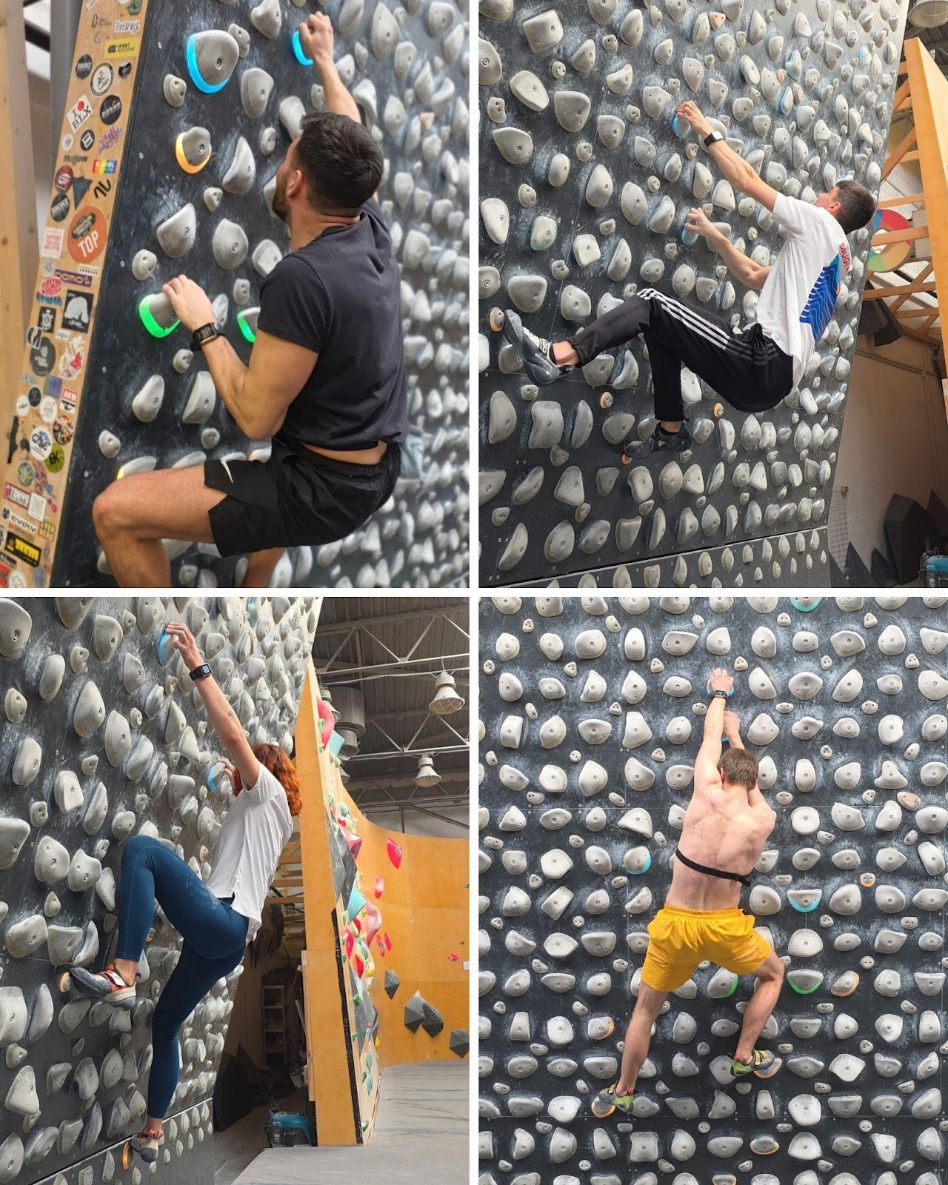


## Supplementary Tables

# Supplemental Table 1: Feasibility Outcomes.

| **Domain** | **Benchmark** | **Achieved** | **Details** | **Rationale** |
| --- | --- | --- | --- | --- |
| **Recruitment & Safety** | | | | |
| Recruitment efficiency | ≥70% | 74.5% (38/51) | 13 ineligible at screening | Conservative threshold for specialized athletic populations (Thabane et al., 2010) (Teresi, J.A., et al. 2022) |
| Protocol completion | ≥85% | 93,7% (30/32) | 2 incomplete datasets | Exceeds standard pilot study benchmarks (Teresi, J.A., et al. 2022) |
| Serious adverse events | 0% | 0% | No injuries or medical events | Exercise intervention safety (Niemeijer et al. 2020) |
| Minor adverse events | - | 10.7% (3/28) | Transient, self-resolved |  |
| **Protocol Validity** | | | | |
| Protocol fidelity | ≥90% | 96.4% (27/28) | 1 required easier route | High fidelity required for standardized fatigue induction (Mihee An et al. 2020) |
| Perceived exertion | ≤16 (RPE) | 14.7 ± 1.8 | "Somewhat hard to hard" | Acceptable training intensity; "somewhat hard to hard" range (Borg, G.A. (1982) (Peterman et al. 20203) |
| Target fatigue achieved | ≥80% | 75% (forearm fatigue) | Forearm fatigue primary | Sport-specific termination validates ecological validity (MacDonalnd et al. 2022), |
| Physiological demand | ≥85% HRmax | 89.3% HRmax | 178.6 ± 11.1 bpm | 85% HRmax corresponds to vigorous-intensity exercise (Swain 2002) |

| **Variable 1** | **Variable 2** | **Pearson** | | **Spearman** | | **Strength** | **Direction** |
| --- | --- | --- | --- | --- | --- | --- | --- |
|  |  | **r** | **p-value** | **ρ** | **p-value** |  |  |
| HRAVG | HRMAX | 0.854 | < 0.001 | 0.871 | < 0.001 | Very Strong | Positive |
| Ascents | Pftime | 0.796 | < 0.001 | 0.579 | 0.0012 | Very Strong | Positive |
| Borg20 | Borg10 | 0.746 | < 0.001 | 0.749 | < 0.001 | Very Strong | Positive |
| HRSTART | HRMAX | 0.709 | < 0.001 | 0.684 | 0.0001 | Very Strong | Positive |
| HRSTART | HRAVG | 0.631 | 0.0003 | 0.658 | 0.0001 | Strong | Positive |
| Borg20 | Pftime | 0.398 | 0.0361 | 0.446 | 0.0173 | Moderate | Positive |
| HRMAX | Borg20 | -0.330 | 0.0860 | -0.348 | 0.0694 | Moderate | Negative |
| HRSTART | Borg20 | -0.319 | 0.0984 | -0.279 | 0.1498 | Moderate | Negative |
| Borg10 | Pftime | 0.311 | 0.1073 | 0.370 | 0.0529 | Moderate | Positive |
| HRSTART | Ascents | -0.305 | 0.1145 | -0.294 | 0.1290 | Moderate | Negative |
| HRSTART | Pftime | -0.197 | 0.3144 | -0.179 | 0.3634 | Weak | Negative |
| HRAVG | Borg20 | -0.193 | 0.3246 | -0.186 | 0.3439 | Weak | Negative |
| HRAVG | Pftime | -0.147 | 0.4559 | -0.104 | 0.5967 | Weak | Negative |
| HRMAX | Pftime | -0.138 | 0.4848 | -0.032 | 0.8724 | Weak | Negative |
| Borg20 | Ascents | 0.123 | 0.5328 | -0.037 | 0.8516 | Weak | Positive |
| HRAVG | Ascents | -0.099 | 0.6157 | -0.131 | 0.5055 | Negligible | Negative |
| HRSTART | Borg10 | -0.087 | 0.6603 | -0.040 | 0.8407 | Negligible | Negative |
| HRMAX | Borg10 | -0.074 | 0.7096 | -0.176 | 0.3709 | Negligible | Negative |
| HRMAX | Ascents | -0.061 | 0.7582 | 0.037 | 0.8525 | Negligible | Negative |
| HRAVG | Borg10 | -0.037 | 0.8516 | -0.065 | 0.7423 | Negligible | Negative |
| Borg10 | Ascents | 0.015 | 0.9415 | -0.142 | 0.4723 | Negligible | Positive |

**Supplemental Table 2. Correlation Analysis.**

**Note:** Table combines Pearson and Spearman correlation coefficients with their respective p-values. The correlations are sorted by the strength of the Pearson correlation coefficient. Color coding indicates correlation strength and direction, while the left border indicates statistical significance level.

**Supplementary Tables S3A and S3B. Baseline subgroup comparability.**

**Supplemental Table S3A. Baseline characteristics by experience level (Advanced vs Intermediate).**

| **Variable** | **Advanced (n=15) Mean ± SD** | **Advanced Median (IQR)** | **Intermediate (n=13) Mean ± SD** | **Intermediate Median (IQR)** | **p-value** | **ES (g/r)** |
| --- | --- | --- | --- | --- | --- | --- |
| **Anthropometrics** |  |  |  |  |  |  |
| Age (years) | 22.5 ± 4.6 | 23 (19–26) | 25.8 ± 5.4 | 24 (23–31) | 0.103 | −0.63 (g) |
| Body mass (kg) | 71.6 ± 11.1 | 74.2 (64.8–80.5) | 70.4 ± 13.6 | 66.7 (64.4–73.1) | 0.807 | 0.09 (g) |
| Height (cm) | 177.3 ± 6.2 | 180 (171.5–182.2) | 174.2 ± 7.8 | 176 (167–180) | 0.264 | 0.43 (g) |
| BMI (kg/m²) | 22.6 ± 2.4 | 23.2 (21.6–24.2) | 23.0 ± 2.9 | 22.5 (21.6–24.7) | 0.718 | −0.14 (g) |
| **Training exposure** |  |  |  |  |  |  |
| Climbing experience (months) | 38.3 ± 35.2 | 21 (16–51.5) | 26.2 ± 28.3 | 12 (7–29) | 0.106 | 0.36 (r) |
| Training frequency (sessions/wk) | 3.3 ± 0.6 | 3 (3–4) | 2.6 ± 0.8 | 2 (2–3) | 0.015 | 0.51 (r) |
| **Physiological (baseline)** |  |  |  |  |  |  |
| HRstart (bpm) | 119.3 ± 20.4 | 120 (104–129) | 118.3 ± 24.1 | 119 (103–131) | 0.314 | 0.39 (g) |
| HRavg (bpm) | 164.0 ± 13.8 | 161 (157–170) | 162.1 ± 14.9 | 160 (157–169) | 0.563 | 0.22 (g) |
| HRmax (bpm) | 178.2 ± 10.9 | 178 (171–184) | 179.0 ± 11.7 | 178 (171–184) | 0.686 | 0.15 (g) |
| Borg RPE (6–20) | 14.9 ± 1.7 | 15 (14–16) | 14.4 ± 1.9 | 15 (13–16) | 0.387 | 0.20 (r) |
| Borg CR10 (0–10) | 5.9 ± 1.5 | 6 (5–7) | 5.9 ± 1.7 | 6 (5–7) | 0.944 | −0.02 (r) |
| **Performance (baseline)** |  |  |  |  |  |  |
| Completed ascents (n) | 4.7 ± 3.2 | 3 (2–7) | 2.4 ± 1.5 | 2 (1–3) | 0.025 | 0.48 (r) |
| Time to exhaustion (s) | 184.3 ± 104.1 | 166 (122–208) | 146.2 ± 77.4 | 127 (99–171) | 0.357 | 0.21 (r) |

**Supplementary Table S3B**. Baseline characteristics by sex (Male vs Female).

| **Variable** | **Male (n=18) Mean ± SD** | **Male Median (IQR)** | **Female (n=10) Mean ± SD** | **Female Median (IQR)** | **p-value** | **ES (g/r)** |
| --- | --- | --- | --- | --- | --- | --- |
| **Anthropometrics** |  |  |  |  |  |  |
| Age (years) | 23.8 ± 5.2 | 23.5 (20–26) | 24.4 ± 5.3 | 23 (23–27.5) | 0.788 | −0.11 (g) |
| Body mass (kg) | 76.8 ± 10.2 | 77.5 (66.3–82.2) | 60.7 ± 7.6 | 62 (54.9–66.2) | <0.001 | 1.67 (g) |
| Height (cm) | 179.4 ± 5.2 | 180.5 (176.8–182.4) | 169.5 ± 5.1 | 170 (166.2–171) | <0.001 | 1.87 (g) |
| BMI (kg/m²) | 23.8 ± 2.2 | 23.6 (22.5–24.6) | 21.1 ± 2.4 | 21.8 (19.9–22.3) | 0.011 | 1.12 (g) |
| **Training exposure** |  |  |  |  |  |  |
| Climbing experience (months) | 29.6 ± 32.3 | 16.5 (10.5–28.5) | 38.2 ± 33.0 | 23.5 (11.2–60) | 0.501 | −0.16 (r) |
| Training frequency (sessions/wk) | 3.2 ± 0.8 | 3 (3–4) | 2.6 ± 0.5 | 3 (2–3) | 0.043 | 0.44 (r) |
| **Physiological (baseline)** |  |  |  |  |  |  |
| HRstart (bpm) | 120.1 ± 21.1 | 120 (103–130) | 116.9 ± 23.4 | 118 (104–130) | 0.830 | 0.10 (g) |
| HRavg (bpm) | 163.8 ± 13.6 | 161 (158–170) | 162.1 ± 15.5 | 160 (155–169) | 0.904 | −0.05 (g) |
| HRmax (bpm) | 178.4 ± 11.0 | 178 (171–184) | 178.9 ± 11.5 | 179 (171–185) | 0.795 | −0.10 (g) |
| Borg RPE (6–20) | 14.8 ± 1.7 | 15 (14–16) | 14.5 ± 2.0 | 15 (13–16) | 0.465 | 0.17 (r) |
| Borg CR10 (0–10) | 5.9 ± 1.6 | 6 (5–7) | 5.8 ± 1.7 | 6 (5–7) | 0.642 | 0.11 (r) |
| **Performance (baseline)** |  |  |  |  |  |  |
| Completed ascents (n) | 4.2 ± 3.1 | 3 (2–6) | 2.6 ± 2.3 | 2 (1–3) | 0.201 | 0.29 (r) |
| Time to exhaustion (s) | 175.8 ± 98.2 | 152 (113–202) | 150.3 ± 82.1 | 129 (104–171) | 0.581 | 0.13 (r) |

Note. Data are presented as Mean ± SD and Median (IQR). Hedges g is reported for parametric comparisons and rank-biserial r for non-parametric comparisons. Sex-based anthropometric differences reflect normal biological dimorphism, and higher training frequency in advanced climbers reflects expected exposure differences. Physiological load at task onset (HR/RPE) did not differ meaningfully between subgroups, confirming matching effectiveness.

**Supplementary Table S4A**. PRE–POST secondary outcomes by experience level (Advanced vs Intermediate)

| **Outcome (unit)** | **Intermediate (n=13) PRE (Mean±SD)** | **Intermediate POST (Mean±SD)** | **Δ (%)** | **dz [95% CI]** | **p** | **Advanced (n=15) PRE (Mean±SD)** | **Advanced POST (Mean±SD)** | **Δ (%)** | **dz [95% CI]** | **p** |
| --- | --- | --- | --- | --- | --- | --- | --- | --- | --- | --- |
| Pinch Strength (kg) | 17.42±2.39 | 16.28±2.05 | −6.5% | −0.55 [−1.20, 0.10] | 0.071 | 16.82±2.80 | 15.95±2.56 | −5.2% | −0.50 [−1.09, 0.09] | 0.073 |
| Stork Balance (s) | 6.13±2.96 | 8.01±7.39 | +30.7% | 0.35 [−0.27, 0.98] | 0.228 | 8.56±5.82 | 11.17±12.34 | +30.5% | 0.28 [−0.29, 0.84] | 0.298 |
| Y-Balance Composite (%) | 98.05±5.91 | 96.71±8.13 | −1.4% | −0.29 [−0.91, 0.33] | 0.317 | 101.08±7.28 | 101.24±6.84 | +0.2% | 0.06 [−0.50, 0.61] | 0.835 |
| Foot Rise (%) | 79.09±5.59 | 81.58±5.71 | +3.1% | 0.48 [−0.16, 1.12] | 0.110 | 83.82±7.54 | 84.64±7.90 | +1.0% | 0.34 [−0.23, 0.91] | 0.214 |
| Finger Hang Time (s) | 14.31±9.73 | 8.40±7.09 | −41.3% | −0.84 [−1.54, −0.14] | 0.010 | 33.27±15.97 | 22.78±9.19 | −31.5% | −0.91 [−1.57, −0.25] | 0.003 |
| Power Slap (% arm length) | 54.32±28.08 | 49.83±25.04 | −8.3% | −0.55 [−1.19, 0.10] | 0.073 | 86.29±20.28 | 83.84±20.02 | −2.8% | −0.52 [−1.12, 0.07] | 0.062 |
| Corsi Block Test (points) | 54.92±24.43 | 73.15±27.69 | +33.2% | 1.05 [0.30, 1.80] | 0.003 | 68.47±30.11 | 71.67±26.08 | +4.7% | 0.18 [−0.38, 0.74] | 0.491 |

**Supplementary Table S4B**. PRE–POST secondary outcomes by sex (Male vs Female).

| **Outcome (unit)** | **Male (n=18) PRE (Mean±SD)** | **Male POST (Mean±SD)** | **Δ (%)** | **dz [95% CI]** | **p** | **Female (n=10) PRE (Mean±SD)** | **Female POST (Mean±SD)** | **Δ (%)** | **dz [95% CI]** | **p** |
| --- | --- | --- | --- | --- | --- | --- | --- | --- | --- | --- |
| Pinch Strength (kg) | 17.33±3.02 | 16.05±2.37 | −7.4% | −0.71 [−1.26, −0.15] | 0.008 | 16.69±1.62 | 16.21±2.30 | −2.9% | −0.24 [−0.97, 0.48] | 0.460 |
| Stork Balance (s) | 7.19±4.07 | 9.38±11.13 | +30.4% | 0.26 [−0.25, 0.76] | 0.288 | 7.87±6.11 | 10.29±9.11 | +30.7% | 0.39 [−0.35, 1.13] | 0.249 |
| Y-Balance Composite (%) | 99.93±6.87 | 99.44±7.00 | −0.5% | −0.18 [−0.68, 0.32] | 0.460 | 99.21±6.82 | 98.59±9.15 | −0.6% | −0.12 [−0.83, 0.60] | 0.725 |
| Foot Rise (%) | 79.70±6.50 | 81.03±6.36 | +1.7% | 0.42 [−0.10, 0.93] | 0.096 | 85.09±6.85 | 87.16±6.68 | +2.4% | 0.39 [−0.35, 1.14] | 0.245 |
| Finger Hang Time (s) | 26.63±14.30 | 17.36±10.97 | −34.8% | −0.98 [−1.58, −0.37] | 0.001 | 20.58±19.72 | 13.84±11.07 | −32.8% | −0.64 [−1.42, 0.15] | 0.075 |
| Power Slap (% arm length) | 83.93±20.46 | 81.10±20.08 | −3.4% | −0.49 [−1.02, 0.04] | 0.053 | 48.98±28.61 | 44.56±25.29 | −9.0% | −0.56 [−1.33, 0.21] | 0.112 |
| Corsi Block Test (points) | 62.11±27.83 | 72.17±23.74 | +16.2% | 0.48 [−0.04, 1.01] | 0.057 | 62.30±29.76 | 72.70±31.88 | +16.7% | 0.68 [−0.11, 1.47] | 0.060 |

Notes. Δ (%) expresses the percent change from PRE to POST. Subgroup analyses are descriptive/exploratory and were not modelled as TIME×GROUP interactions.
